# Combination of carbonic anhydrase inhibitor, acetazolamide, and sulforaphane, reduces the viability and growth of bronchial carcinoid cell lines

**DOI:** 10.1186/1471-2407-13-378

**Published:** 2013-08-08

**Authors:** Reza Bayat Mokhtari, Sushil Kumar, Syed S Islam, Mehrdad Yazdanpanah, Khosrow Adeli, Ernest Cutz, Herman Yeger

**Affiliations:** 1 Developmental and Stem Cell Biology, University of Toronto, Toronto, ON, Canada; 2 Division of Haematology/Oncology, The Hospital for Sick Children and University of Toronto, Toronto, ON, Canada; 3 Department of Paediatric Laboratory Medicine, The Hospital for Sick Children, Institute of Medical Science, University of Toronto, Toronto, ON, Canada; 4 Division of Urology, The Hospital for Sick Children, University of Toronto, Toronto, ON, Canada; 5 Department of Laboratory Medicine & Pathobiology, University of Toronto, Toronto, ON, Canada

**Keywords:** Bronchial carcinoids, Pulmonary neuroendocrine tumor, Serotonin, Carbonic anhydrase, Acetazolamide, Sulforaphane

## Abstract

**Background:**

Bronchial carcinoids are pulmonary neuroendocrine cell-derived tumors comprising typical (TC) and atypical (AC) malignant phenotypes. The 5-year survival rate in metastatic carcinoid, despite multiple current therapies, is 14-25%. Hence, we are testing novel therapies that can affect the proliferation and survival of bronchial carcinoids.

**Methods:**

In vitro studies were used for the dose–response (AlamarBlue) effects of acetazolamide (AZ) and sulforaphane (SFN) on clonogenicity, serotonin-induced growth effect and serotonin content (LC-MS) on H-727 (TC) and H-720 (AC) bronchial carcinoid cell lines and their derived NOD/SCID mice subcutaneous xenografts. Tumor ultra structure was studied by electron microscopy. Invasive fraction of the tumors was determined by matrigel invasion assay. Immunohistochemistry was conducted to study the effect of treatment(s) on proliferation (Ki67, phospho histone-H3) and neuroendocrine phenotype (chromogranin-A, tryptophan hydroxylase).

**Results:**

Both compounds significantly reduced cell viability and colony formation in a dose-dependent manner (0–80 μM, 48 hours and 7 days) in H-727 and H-720 cell lines. Treatment of H-727 and H-720 subcutaneous xenografts in NOD/SCID mice with the combination of AZ + SFN for two weeks demonstrated highly significant growth inhibition and reduction of 5-HT content and reduced the invasive capacity of H-727 tumor cells. In terms of the tumor ultra structure, a marked reduction in secretory vesicles correlated with the decrease in 5-HT content.

**Conclusions:**

The combination of AZ and SFN was more effective than either single agent. Since the effective doses are well within clinical range and bioavailability, our results suggest a potential new therapeutic strategy for the treatment of bronchial carcinoids.

## Background

Bronchial carcinoid tumors are a group of neuroendocrine tumors (NETs), which constitute roughly 1–2% of all lung malignancies in the adult population and account for 31% of all cases of carcinoids [1]. These tumors are classified as typical (TC) and atypical (AC). The 5-year survival rate is 98% for TC and 76% for AC [2]. Furthermore, it is thought that tumor-derived 5 hydroxytryptamine (5-HT), or serotonin, causes carcinoid syndrome manifested by skin flushing, excessive diarrhea, right-sided heart disease and bronchoconstriction. Nearly 95% of patients present with right-sided heart valve disease and are associated with poor long-term survival, with death occurring in approximately one-third of these patients. Patients with liver metastases may develop malignant carcinoid syndrome, releasing vasoactive substances into the systemic circulation. Currently, severe carcinoid syndrome is effectively managed with octreotide and lanreotide, which are somatostatin analogs [3]. However, metastatic bronchial carcinoids are incurable and the 5-year survival rate is 20-30% [4]. Conventional cytotoxic agents such as fluorouracil, doxorubicin and cyclophosphamide, which are effective in the treatment of other neoplasms, have been ineffective against carcinoids [5]. Therefore, strategies that target the survival pathways of pulmonary carcinoids are being considered to treat carcinoids. In the present study, we have investigated the efficacies of two drugs, acetazolamide (AZ) and sulforaphane (SFN), which are known to target the survival pathways in other cancers.

AZ is a classic pan-carbonic anhydrases (CAs) inhibitor. CAs help tumor cells to cope with acidic and hypoxic stress by reversible hydration of carbon dioxide to proton and bicarbonate [6], thereby maintaining physiological intracellular pH, despite the acidic extracellular environment. The overexpression of CAs has been reported in a wide variety of human neoplasms and is associated with poor prognosis in many types of cancers, such as breast adenocarcinoma and bladder carcinoma [7,8]. High expressions of HIF-1α and CAs have been reported in ileal carcinoids [9]. Since CAs are a major component of survival pathways of tumor cells, the inhibition of enzymatic activity of CAs has been studied extensively as a therapeutic strategy against cancer [10]. Chemical inhibitors of CAs (CAIs) such as AZ and AZ-based new compounds as single agent or combination therapy with synthesized aromatic sulfonamides such as 2-(4-sulfamoylphe- nyl-amino)-4,6-dichloro-1, 3, 5-triazine (TR1) and 4-[3-(N, N-dimethylaminopropyl) thioreidophenylsulfonylaminoethyl] benzenesulfonamide (GA15) with high affinity for CA9 have been shown to inhibit CA9 enzymatic activity and suppress the invasive capacity, decrease cell proliferation and induce apoptosis in human renal carcinoma and cervical cancer cells [11,12].

5-HT is another crucial factor contributing to the development of NETs, including human pancreatic carcinoid cells [13]. Previous studies have demonstrated that 5-HT stimulates the proliferation of lung carcinoid cell lines [14] and it can function as an autocrine growth factor for carcinoids (and NETs) [14]. We have proved that hypoxia stimulates the release of 5-HT from neuroepithelial bodies, the precursor cells of bronchial carcinoids, and that the blockade of 5-HT3 receptor inhibits hypoxia-induced 5-HT release [15]. We investigated whether our treatments could reduce the production of 5-HT in the tumors, this being relevant to the pathophysiology of the carcinoid syndrome and auto regulatory growth. The inhibition of CAs, which regulate intracellular and extracellular pH, can severely abrogate homeostatic and neuroendocrine functions [16,17]. Previously, the inhibitory effects of AZ on 5-HT secretion and proliferation in rabbit conjunctival epithelium and human renal carcinoma cells have been reported [12,16,17]. Therefore, we hypothesize that AZ will down regulate the secretion of 5-HT and reduce cell viability.

Furthermore, we reasoned that combinatorial treatment of CA inhibitors with other agents that target survival pathways would enhance the efficacy of AZ. In this regard, SFN, known to demonstrate anticancer properties by several mechanisms, is a reasonable candidate. The anticancer mechanisms of SFN include the inhibition of survival pathways, induction of proapoptotic pathways, inhibition of histone deacetylases (HDAC) and induction of Phase-II antioxidant enzymes. The oncogenic pathways affected by SFN are Akt (ovarian cancer) and Wnt/beta catenin (breast cancer) [18-20], whereas, beta catenin accumulation in gastro-intestinal carcinoid cells and the role of PI3K/Akt signaling in pulmonary carcinoids have been established [20,21]. SFN is reported to affect survival pathway by hyperphosphorylation of Rb protein (anti-apoptotic in un-phosphorylated form) in colon cancer cells, and has inhibited cyclin D1 in pancreatic cancer cells [22,23], whereas, cyclin D1-induced Rb overexpression has been found to be upregulated in pulmonary carcinoids [24]. SFN is also an inhibitor of HDAC [25], and other HDAC inhibitors such as valproic acid and suberoyl bis-hydroxamic acid in combination with lithium have demonstrated significant growth inhibition and cell cycle arrest in H-727 cells [26]. SFN has demonstrated synergistic activity with cytotoxic agents (5-fluorouracil, paclitaxel), phytochemicals (resveratol) and targeted therapies (sorafenib, imatinib) [27-30].

In terms of the involvement of 5-HT in bronchial carcinoids, SFN can be an appropriate agent for carcinoid therapy as it has been reported to reduce the expression of 5-HT receptors including 5-HT2, 5-HT3 and serotonin transporter (SERT) as well as to affect the release of 5-HT in Caco-2 cells [31]. We believe that SFN can potentially demonstrate antitumor activity and demonstrate an additive or synergistic effect with AZ in pulmonary carcinoids given the (1) findings that SFN, in other cancers, can target survival pathways which also contribute to the survival and progression of carcinoids, (2) effect of SFN on 5-HT pathway, and (3) the synergistic activity of SFN with other anticancer agents. Since both AZ and SFN can potentially affect the survival mechanisms of pulmonary carcinoids by different mechanisms, we hypothesize that the combination of these two compounds can demonstrate additive or synergistic effect against pulmonary carcinoids. Since SFN down regulates the expression of 5-HT receptors [31], the combination of AZ + SFN might be able to shut down 5-HT-mediated autocrine growth of carcinoid cells.

In the present study, we report our finding that both AZ and/or SFN have inherent antitumor activity and the combination of these agents demonstrates significantly higher antitumor activity in in vitro and in vivo models of bronchial carcinoid (BC).

## Methods

### Drug, reagents and supplements

Acetazolamide (AZ), dimethyl sulfoxide (DMSO), serotonin hydrochloride (5-HT), D4-serotonin, 5-Hydroxyindole-3-acetic acid (5-HIAA) and trans-2-phenylcyclopropylamine hydrochloride were obtained from Sigma-Aldrich (Oakville, ON, Canada). Sulforaphane (SFN) was purchased from LKT Laboratories (St. Paul, MN, USA). RPMI-1640 and EMEM medias, fetal bovine serum (FBS) and penicillin-streptomycin, were purchased from Gibco (Burlington, ON, Canada) and bovine serum albumin (BSA) was obtained from Invitrogen (Grand Island, NY, USA). Matrigel was purchased from BD Biosciences company (La Jolla, CA, USA). Methylcellulose was obtained from MethoCult company (Vancouver, BC, Canada). Phosphate-buffered Saline (PBS) was purchased from Multicell (St. Bruno, QC, Canada).

### Cell lines

The lung carcinoid cell lines, well differentiated H-727 (TC) and poorly differentiated H-720 (AC), were purchased from the American Type Culture Collection (ATCC). Fetal lung fibroblast (FLF) strain, available in our cell bank was used as a normal control.

### Cell culture

The lung carcinoid and fetal lung fibroblast cell lines were maintained in RPMI-1640 and EMEM, respectively. The medias were supplemented with 10% heat-inactivated FBS, 100 IU/ml and penicillin, 100 ug/ml streptomycin at 37.0°C, 5% CO_2_. We tested the effect of varying concentrations of FBS (0-20%) on the proliferation of H-727 and H-720 cells to determine the minimum percentage of FBS needed for cell survival for an experiment of 7 days. The cells were plated in 48-well black walled plates (Falcon) at 20,000 cells/well and incubated overnight (37°C and 5% CO_2_). Fresh supplemented media including the different percentages (1-20%) of FBS were added every other day for a period of seven days.

### Animals

Four-to-six-week-old female NOD/SCID mice were obtained from the animal facility at The Hospital for Sick Children (SickKids) and used for our in vivo study within the guidelines of the Lab Animal Services. The protocols for animal experimentation were approved by the Animal Safety Committee, Sickkids Research Institute.

### Trypan blue exclusion assay

Trypan blue exclusion assay was used to assess cell viability. Following the indicated treatments, cells were trypsinized and incubated with trypan blue (Multicell, Wisent Inc. St. Bruno, QC, Canada) (final volume 20% added to media) for 10 minutes at 37°C. Percent viability was calculated as the number of trypan blue positive per total cells counted per microscopic field (total of 4 fields per condition).

### AlamarBlue cytotoxicity assay

Cells were seeded (5,000 and 20,000 cells) in 48-well plates in complete medium. After 48 hours, cells were treated with AZ and/or SFN for 48 hours and 7 days. The highest concentration of DMSO (2 × 10^-4^) was used as the vehicle control. AlamarBlue (AbD Serotec, MorphoSys, Raleigh, NC, USA) agent (10% of total volume) was added to each well for 4 hours before fluorometric detection. Fluorescence was measured using the SPECTRAmax Gemini Spectrophotometer at excitation wavelength of 540 nm and emission wavelength of 590 nm. Percent survival vs. control is reported as the mean +/− standard deviation.

### Effect of 5-HT on growth of lung carcinoid cells

AlamarBlue assay was performed to determine whether AZ and/or SFN could block the effects of 5-HT on H-727 and H-720 growth. Cells were treated for 7 days with AZ and/or SFN (0–80 μM) after adding 5-HT exogenously (0.01 nM for H-727 and 10 nM for H-720) into the supplemented media (2.5% FBS). Trans-2-phenylcyclopropylamine hydrochloride, a monoamine oxidase inhibitor (MAOI), (2 μM) was added to prevent metabolism of 5-HT during the experiment [32,33].

### Matrigel invasion assay

Invasion assay was performed as previously described [34]. Eight um pore size polyvinyl membrane-based chambers (Corning Life Sciences, Lowell, MA, USA) were coated with 100 μl of ice-cold matrigel. The matrigel-coated chambers were incubated at 37°C for 4 hours, after which 30,000 cells were added to the upper chamber. Five hundred μl RPMI-1640 media were filled in the lower chamber. The whole system was incubated at 37°C for 24 hours. The top part of the incubated chamber was then removed and invading cells were counted following crystal violet staining.

### Methylcellulose clonogenic assay

H-727 and H-720 cells were treated with varying concentrations (10 μM, 20 μM and 40 μM) of AZ and/or SFN in a medium supplemented by 10% FBS for 7 days every other 48 hours. To assess the clonogenic potential of treated cells, at the end of the seventh day, cells were trypsinized and resuspended (3 × 10^4^ cells/ml) in 40% methylcellulose supplemented with RPMI-1640, 10% FBS and 1% antibiotics (100 IU/ml penicillin and 100 μg/ml streptomycin) and plated in 35 mm tissue culture dishes (Nalgene Nunc International, Rochester, NY, USA) in triplicate and incubated in 5% CO2 at 37°C. After two weeks, the numbers of colonies were counted by using a grading dish on a phase contrast microscope (×10). Clonogenicity was determined as the average of number of colonies per dish for each treatment group.

### In vivo efficacy of AZ and SFN

H-727 and H-720 cells (2 × 10^6^) were injected into the subcutaneous inguinal fat pad of NOD/SCID mice. When the tumors attained a diameter of 0.5 cm, the mice were randomized into 4 groups (5 mice per group). The control and treatment groups received intrapertoneal injections of either vehicle (PBS) or AZ (20 mg/kg) and/or SFN (40 mg/kg), respectively, every day for two weeks. Experiment was terminated when tumor sizes exceeded 2 cm^2^ in diameter or animals showed signs of morbidity. Tumor diameters were measured on a daily basis until termination. The long (D) and short diameters (d) were measured with calipers. Tumor volume (cm^3^) was calculated as V = 0.5 × D × d^2^. After euthanizing the mice, the tumors were resected, weighted and fixed in 10% neutral-buffered formalin at room temperature and processed for histopathology.

### Electron microscopic analysis

Tumor fragments were fixed in 4% formaldehyde and 1% glutaraldehyde in phosphate buffer, pH 7.4, and post fixed in 1% osmium tetroxide. Tumor tissues were then dehydrated in a graded series of acetone from 50 to 100% and subsequently infiltrated and embedded in Epon-Araldite epoxy resin. The processing steps from post fixation to polymerization of resin blocks were carried out in a microwave oven, Pelco Bio Wave 34770 (Pelco International, Clovis, CA, USA) using similar procedures but with a slight modification as recommended by the manufacturer. Ultrathin sections were cut with a diamond knife on the Reichert Ultracut E (Leica Inc., Vienna, Austria). Sections were stained with uranyl acetate and lead citrate before being examined in the JEM-1011 (JEOL USA, Inc., Peabody, MA, USA). Digital electron micrographs were acquired directly with a 1024 × 1024 pixels CCD camera system (AMT Corp., Danvers, MA, USA) attached to the ETM (1200 EX electron microscope).

### Immunofluorescence methods

Frozen sections (5 μm) were immersed in precooled acetone at −20°C for 10 minutes and allowed to dry at room temperature for 20 minutes; sections were washed in double distilled water. Antigen retrieval was performed by heating in a microwave for 14 minutes in tri-sodium citrate buffer (pH 6.0). To block non-specific binding, sections were treated with 4% BSA for 30 minutes. The sections were incubated with primary antibodies at 4°C overnight. The primary antibodies used as follow: anti-chromogranin A (Dako, Carpinteria, CA, USA), anti-ki67 (Dako, Carpinteria, CA, USA) and anti-phospho-Histone H3 (Temecula, CA, USA). After this overnight incubation, primary antibodies incubation sections were washed with PBS 3 × 10 minutes each at RT and bound primary antibodies were detected using secondary antibodies diluted in 4% BSA. Sections were incubated for 1 hour in secondary antibody-donkey anti-goat (Abcam, Cambridge, MA, USA) and chicken anti-rabbit (Invitrogen, Grand Island, NY, USA) at RT. Finally, sections were washed in PBS 3 × 10 minutes each and mounted with VectaShield (Dako, Carpinteria, CA, USA) mounting medium with DAPI (4′,6-diamidino-2-phenylindole; Sigma-Aldrich, St. Louis, MO, USA). For negative control, sections were incubated in secondary antibodies only. Mounted slides were visualized using a fluorescence microscope at × 10 and × 40 magnification (Nikon DXM1200 digital camera, NortonEclipse software version 6.1). For quantification, the percentage of positive cells was calculated using the formula [X (6 low power fields of positive staining)/Y (total count per 6 fields) × 100]. The level of immunofluorescence (IF) of the positive cells was also examined by ImageJ64 software.

### Immunohistochemistry

Immunohistochemistry (IHC) was performed on paraffin sections as previously described [35]. After deparaffinization through xylene and graded alcohols into water and rehydration in water, slides were antigen retrieved in 10 mM sodium citrate buffer (pH 6.0) by heating in a microwave oven for 10 minutes. After cooling the sections for 20 minutes at room temperature, endogenous peroxidase activity was blocked by incubation with 3% hydrogen peroxide in methanol for 10 minutes. After washing in PBS (pH 7.4) for a further 5 minutes and blocking non-specific binding by incubating in 3% BSA/PBS for 10 minutes, the sections were incubated with monoclonal mouse anti-human Ki-67 antigen/FITC (MIB-1);(1:50) (DakoCytomation, Glostrup, Denmark) at 4°C overnight. Afterwards, the slides were washed several times with PBS and incubated at room temperature with a broad-spectrum poly horseradish peroxidase (HRP) conjugate as a secondary antibody (Invitrogen, Zymed, Burlington, ON, Canada). Next, the slides were washed with PBS several times and stained with DAB (3, 3′-diaminobenzidine; Vector Laboratories, Orton Southgate, Peterborough, United Kingdom) for two minutes. After washing again with PBS, the slides were then stained with hematoxylin and mounted. Negative controls included incubation in the relevant secondary antibodies only.

### Measurement of 5-HT content

To assess the cellular and plasma content of 5-HT and its metabolite, 5-Hydroxyindoleacetic acid (5-HIAA), we used a sensitive Liquid Chromatography-Mass Spectrometry (LC-MS) method as follows. Samples consisting of calibrators, Quality control (QC), cell pellet or tissue homogenate were spiked with 2 nm of d4-serotonin. The mixtures were applied to a Centri-Free centrifugal filter unit (30,000 MWCO) and centrifuged at 1000 g for 30 minutes. To 500 μL of calibrator, cell pellet or tissue homogenate 20 μL of d_4_-5-HT solution (100 μM) was added. Each sample mixture was vortex-mixed and transferred to a Centri-Free centrifugal filter unit (30,000 MWCO) and centrifuged at 1000 g for 30 minutes. The filtrates were transferred to HPLC auto-sampler vials and a 1 μL aliquot was analyzed by LC-MS. The LC-MS system consisted of an API4000 QTRAP mass spectrometer (Applied Biosystems Inc. Foster, CA, USA) and an Agilent 1200 series HPLC (Agilent Technologies, USA). 5-HT and 5-HIAA were separated on an Agilent Eclipse XDB C18 column (100 × 4.6 mm, 1.8 mm). High Performance Liq-Chromatography (HPLC) mobile phase consisted of A: 2 mmol/L ammonium formate in H_2_O + 0.1% formic acid and B: 2 mmol/L ammonium formate in methanol + 0.1% formic acid. The HPLC flow rate was 800 μL/min and the chromatographic gradient consisted of 90% A increasing to 100% B in 5 minutes. The mobile phase composition was kept at 100% B for 2 minutes and subsequently the column was equilibrated with 90% A for 3 minutes. The mass spectrometry was conducted in positive electrospray ionization mode. The ion transitions of 177.1 → 160.1 m/z, 181.2 → 164.1 m/z, and 192.1 → 146.1 m/z were monitored for the detection and quantitation of 5-HT, D_4_-5-HT and 5-HIAA, respectively. The dwell time for each ion transition was set to 100 msec. The de-clustering potential and collision energy for 5-HT and D_4_-5-HT was set to 36 and 15, and for 5-HIAA at 65 and 20. Data analysis and analyte quantification was performed using the Analyst software Auto-Quant feature. The unknown analyte signal was measured against the calibration curve to obtain the concentration values.

### Statistical analysis

Graphing and statistical analysis were performed with Graph Pad. Unpaired Student’s *t*-Test and ANOVA software were used to obtain the test of significance and in all analysis the significance levels were specified at p ≤ 0.05 (*), p ≤ 0.01 (**), p ≤ 0.001 (***) and p ≤ 0.0001 (****). All in vitro experiments were done in triplicate.

## Results

### Dose-dependent inhibition of growth of lung carcinoid and fetal lung fibroblast cell lines with AZ and/or SFN treatment alone

**Figure 1:**
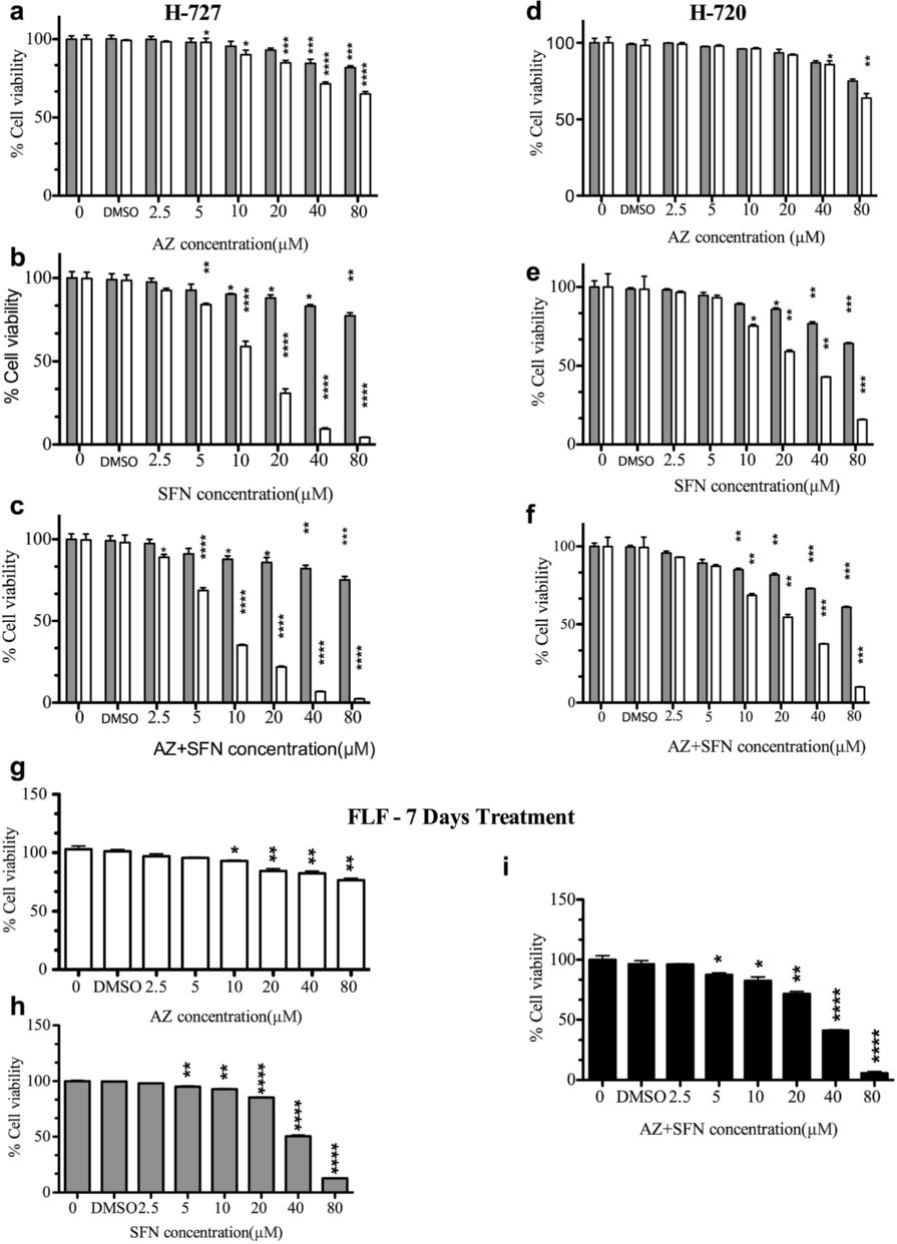
**AZ and/or SFN Treatment Inhibit Growth of Lung Carcinoid and Fetal Lung Fibroblast Cells (H-727 and H-720); 48 hours, 7 days) and (FLF): AlamarBlue assay; dose response for AZ, SFN and AZ + SFN; (0-80 μM) treatment in H-727 (a,b and c), H-720 (d, e and f) cells 48 hours (gray) and 7 days (white) and AlamarBlue assay; dose response for AZ (white), SFN (gray) and AZ + SFN (black); (0-80 μM) treatment in FLF (g, h and i) cells, 7 days.** The significance level compared to control (p value) was specified as follows: *(p < 0.05), **(p < 0.01), ***(p < 0.001) and ****(p < 0.0001).

To determine the effect of AZ and/or SFN treatment on the growth of H-727 and H-720 cells, AlamarBlue assay was performed. Both AZ and SFN showed a dose-dependent inhibitory effect on H-727 and H-720 cells. Significant growth inhibition of H-727 cells was obtained after treatment with 40 μM AZ for 48 h. In the case of SFN, 10 μM concentration caused significant reduction in growth inhibition of H-727. Whereas 48 h treatment with AZ did not affect the viability of H-720 at any of the concentrations, SFN caused significant inhibitory effect on H-720 at 10 μM after 48 h treatment. After 7 days of treatment, a significant reduction of viability was seen in H-727 cells and H-720 cells. SFN at the concentrations of 5 μM and 10 μM had significant inhibitory effect after 7 days of treatment on H-727 and H-720, respectively. In comparison to single agents, the combination of AZ and SFN produced a significant reduction in viability of H-727 and H-720 cells at a lower concentration. After 48 hours, a significant reduction in viability was seen with a combination of 10 μM of both AZ and SFN in H-727 and H-720 cells. Seven days of treatment with 2.5 μM and 10 μM AZ and SFN caused significant reduction in cell viability of H-727 and H-720 cells, respectively (Figure 1a-f and Table 1). Additionally, IC50 decreased in both single and combination therapy in H-727 cells (AZ: 117 μM, SFN: 11 μM and AZ + SFN: 7 μM) and H-720 cells (AZ: 166 μM, SFN: 25 μM and AZ + SFN: 18 μM) after 7 days of treatment. The greater decrease in IC50 for AZ + SFN combination (1.67 fold in H-727 and 1.35 fold H-720) suggests the potentiation of SFN effect by AZ (Figure 1, Table 1). The IC50 of our drugs on normal cells FLF after 7 days of treatment was 514.4 μM, 39.54 μM and 29.68 μM for AZ, SFN and AZ + SFN, respectively. A significant reduction of viability of FLF cells was seen after 7 days of treatment with 10 μM AZ, 5 μM SFN and 5 μM AZ + SFN (Figure 1g-1 and Table 1).

**Table 1:** Dose-dependent inhibition of growth of lung Carcinoid cell lines with AZ and/or SFN treatment alone (Short Term (48 hours) and long term (7 days)

	**Short term**			**Long term**			
**Cell line**	**Drug**		**Concentration (μM); 48 h**	**% Growth inhibition; 48 h**	**Concentration (μM); 7D**	**% Growth inhibition; 7D**	**IC**_**50 **_**(μM); 7D**
H-727	AZ	Min	40	16 +/− 6.2	5	3 +/− 6.4	117.5
		Max	80	19 +/− 6.2	80	35 +/− 3.6	
	SFN	Min	10	10 +/− 1.1	5	17 +/− 2	11.3
		Max	80	23 +/− 7	80	26 +/− 0.4	
	AZ+	Min	10	13 +/− 3.4	2.5	11 +/− 4	6.75
	SFN	Max	80	25 +/− 7	80	98 +/− 0.5	
H-720	AZ	Min	-	-	40	15 +/− 4.1	166.5
		Max	-	-	80	37 +/− 5.1	
	SFN	Min	10	15 +/− 1.6	10	25 +/− 1.6	25.1
		Max	80	36 +/− 1	80	85 +/− 0.5	
	AZ+	Min	10	15 +/− 1.5	10	32 +/− 1.9	18.65
	SFN	Max	80	39 +/− 0.7	80	90 +/− 0.3	
FLF	AZ	Min	-	-	10	8 +/− 1.1	514.4
		Max	-	-	80	24 +/− 2.8	
	SFN	Min	-	-	5	5 +/− 1.2	39.54
		Max	-	-	80	82	
	AZ+	Min	-	-	5	13 +/− 2.6	29.68
	SFN	Max	-	-	80	95 +/− 2.4	

### AZ and/or SFN treatment alone inhibit clonogenic ability of lung carcinoid cell lines

**Figure 2:**
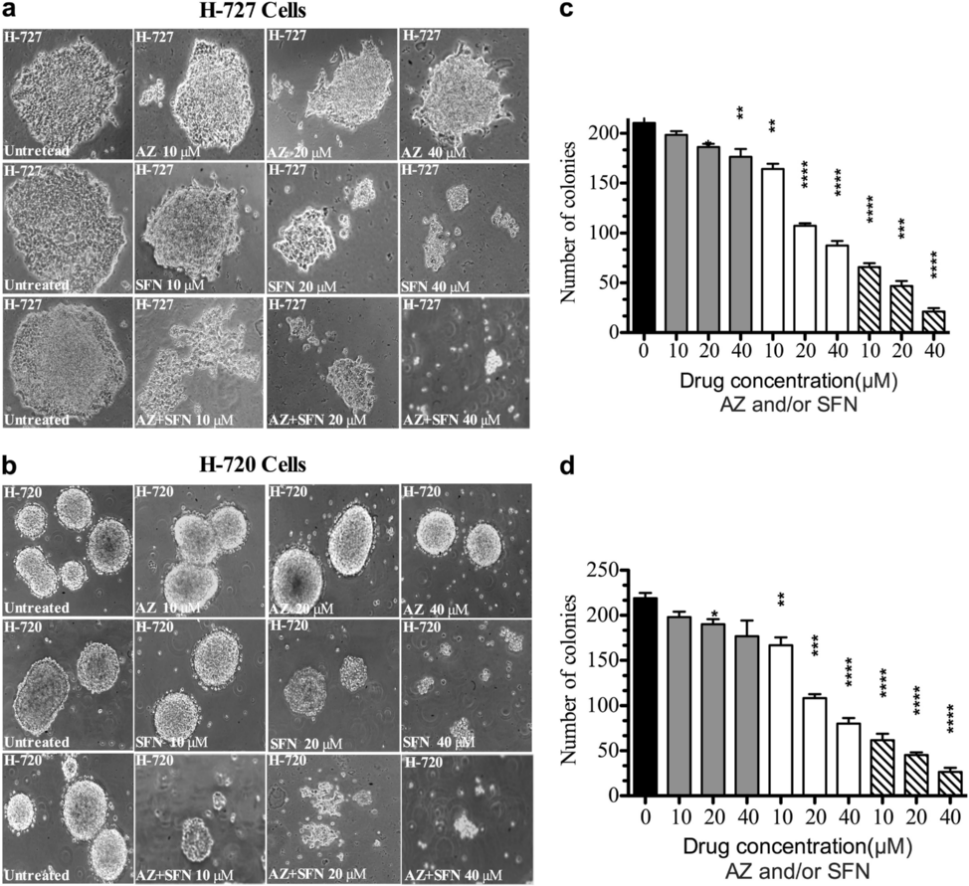
**AZ and/or SFN Treatment Inhibit Clonogenic Ability of Lung Caciniod Cells (H-727 and H-720); (Methylcellulose Clonogenic Assay; 16 Days): (a and b) phase contrast microscopy (× 10) of H-727 and H-720 colonies in Methylcellulose Clonogenic assay; (c and d) figures represent the analysis of Methylcellulose Clonogenic assay for H-727 and H-720 colonies for the untreated (black) and AZ (gray), SFN (white) and AZ + SFN (line pattern), 10 μM, 20 μM and 40 μM; on the day 16th).** The significance level compared to control (p value) was specified as follows: *(p < 0.05), **(p < 0.01), ***(p < 0.001) and ****(p < 0.0001).

To determine the effect of AZ and/or SFN treatment on the clonogenicity of H-727 and H-720 cells, methylcellulose clonogenic assay was performed. H-727 and H-720 cells pre-treated for 7 days with AZ and/or SFN at different concentrations showed a dose-dependent inhibition of colony formation relative to untreated cells in methylcellulose media. Figure 2(a-c) illustrates that the clonogenic capacity of H-727 and H-720 cells cultured in methylcellulose was considerably reduced compared to the control. The minimum concentration of AZ was 20 μM for H-727 (10%; p ≤ 0.05) and H-720 (1%; p ≤ 0.05). The minimum concentration of SFN was 10 μM for H-727 (20%; p ≤ 0.01) and H-720 (2%; p ≤ 0.01). The combination of AZ and SFN significantly reduced clonogenicity, with 10 μM showing significant reduction in clonogenicity of H-727 (65%; p ≤ 0.0001) and H-720 (9%; p ≤ 0.0001) Additionally, the combination treatment resulted in a prominent reduction in the clonogenicity compared to both single agents at 10 μM, 20 μM and 40 μM (p < 0.001) (Figure 2a-c).

### AZ and/or SFN treatment inhibited tumor growth in lung carcinoid cell line xenografts

#### Tumor morphology

**Figure 3:**
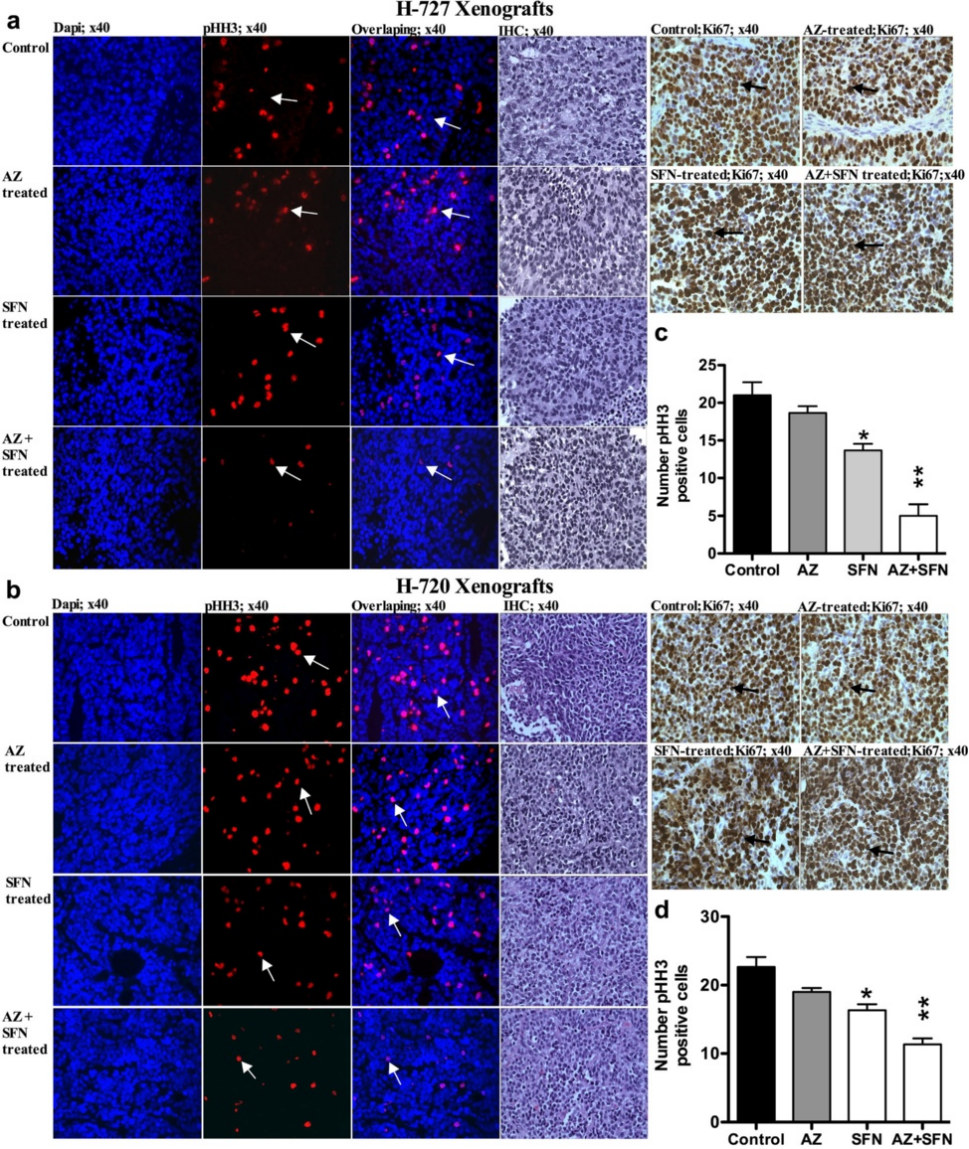
**AZ and SFN Inhibit Tumor Progression in Lung Carcinoid Xenografts: figures (a, b, c, d) and (g, h, i , j, k) represent the volume, morphology, weight, H&E (×10) and ultrastructure (× 10**^**4**^**; nu, represents the nucleous and arrow, represent the cytoplasmic dense-core vesicles) of H-727 and H-720 xenografts, respectively.** Figures **(f and l)** represent the number of dense-core vesicles after treatment with AZ and/or SFN compare to untreated group in H-727 and H-720 xenografts, respectively.

**Figure 4:**
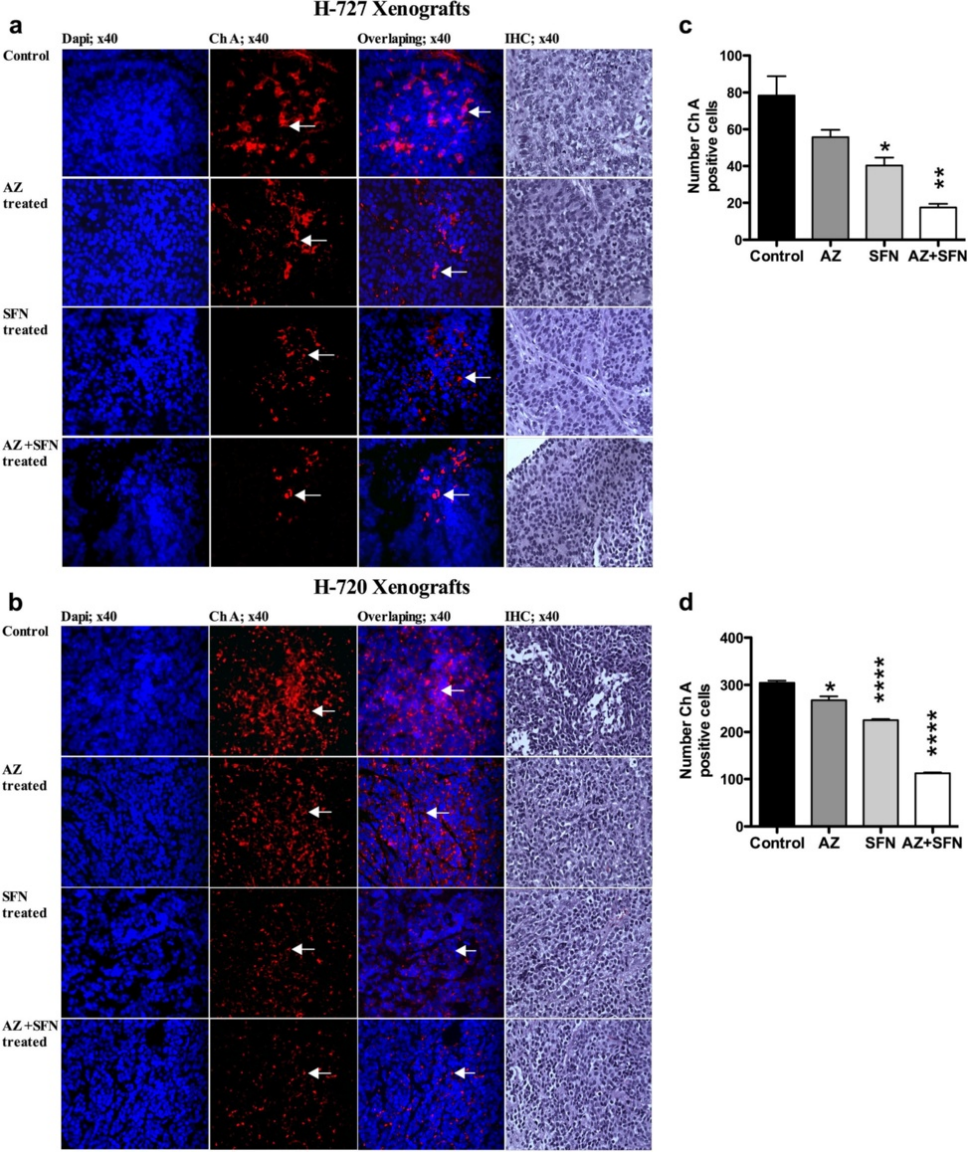
AZ and SFN Treatment Effect on Mitotic Index and Proliferation in Lung Carcinoid Xenografts: figures (a and b) represent the mitotic index (pHH3); (arrows), H&E and proliferation (Ki67) in H-727 and H-720 xenografts, respectively; (c and d) represent the number of pHH3 positive cells for H-727 and H-720 xenografts, respectively, p < 0.05 (*) and p < 0.01(**).

**Figure 5:**
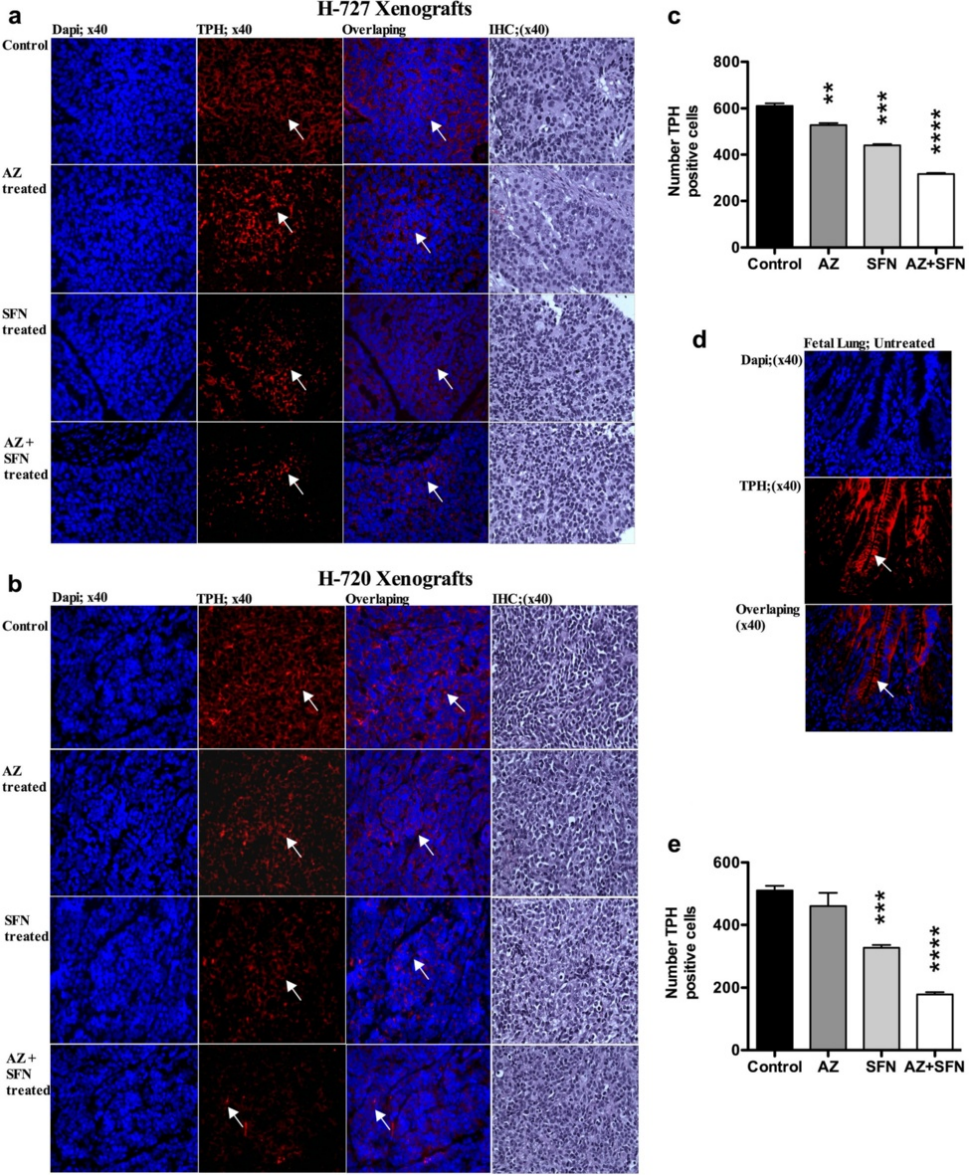
AZ and SFN Treatment Reduce Chromogranin A in Lung Carcinoid Xenografts: figures (a and b) represent Ch A positive cells (arrows) in H-727 and H-720 xenografts, respectively; (c and d) represent the number of Ch A positive cells for H-727 and H-720 xenografts, respectively, p < 0.05 (*), p < 0.01 (**) and p < 0.0001 (****).

**Figure 6:**
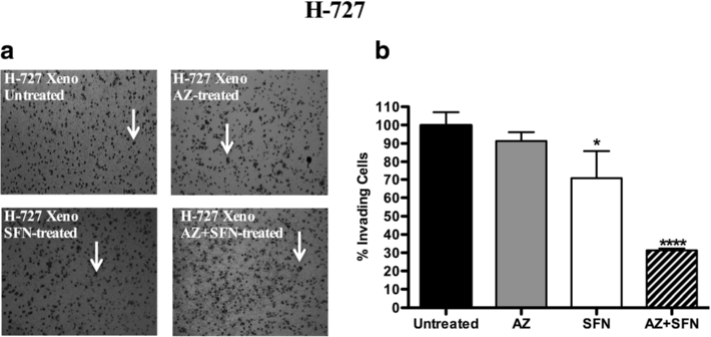
AZ and SFN Trytophan Hydroxylase (TPH) in Lung Carcinoid Xenografts: figures (a and b) represent TPH positive cells (arrow) in H-727 and H-720 xenografts, respectively; (d) represent positive control (fetal lung tissue); (c and e) represent the number of TPH positive cells for H-727 and H-720 xenografts, respectively; p < 0.01 (**), p < 0.001 (***) and p < 0.0001 (****).

In vivo treatment of mice bearing H-727 and H-720 tumors with AZ and/or SFN showed an inhibitory effect on tumor growth. In H-727 xenografts, compared to control, AZ, SFN and AZ + SFN caused 18% (p ≤ 0.05), 35% (p ≤ 0.01) and 73% (p ≤ 0.001) reduction in tumor weights, respectively (Figure 3a, b and c). In H-720 xenografts, AZ, SFN and AZ + SFN caused 4.5%, 41% (p ≤ 0.05) and 65% (p ≤ 0.001) reduction in tumor weights, respectively (Figure 3g, h and i). In H-727 xenografts, the AZ + SFN combination significantly reduced the weight of tumors compared to AZ alone (p < 0.0001). IF results revealed that the number of pHH3 positive cells was reduced significantly in all treatment groups compared to the untreated group, with the AZ + SFN combination inducing 76% (p < 0.0001) and 50% (p < 0.05) reduction in number of pHH3 positive cells in H-727 and H-720 xenografts, respectively. IHC results did not show any change in the number of Ki67 positive cells (Figure 4a-d). IF results showed that the levels of chromogranin A (ChA) (Figure 5a, d) and tryptophan hydroxylase (TPH) (Sigma, Oakville, ON, Canada) (positive controls: fetal lung tissue; Figure 6a-e) reduced significantly in all treatment groups compared to positive controls and untreated groups.

#### Electron microscopy

Electron microscopy of tumor xenografts revealed cells with nuclear fragmentation (nu sign), intact nuclei and cell membrane, and a reduction in cytoplasmic dense-core vesicles (DCV) (arrow sign) in H-727 and H-720 xenografts. In H-727 xenografts, the reduction in the number of DCV was 33%, 58% and 79% for AZ, SFN and AZ + SFN treated groups, respectively. In H-720 xenografts, the reduction in the number of DCV was 24%, 48% and 70% for AZ, SFN and AZ + SFN treated groups, respectively. Compared to the control, AZ, SFN and AZ + SFN significantly reduced the number of granules in treatment groups. AZ + SFN treated tumors had significantly fewer DCV compared to AZ and SFN treated tumors (Figure 3e and k), Table 2.

**Table 2:** The Percentage of Dense-Core Vesicles (DCV) after Treatment with AZ and/or SFN Compare to untreated group in H-727 and H720 xenografts, respectively

**Cell type**	**Treatment**	**% Reduction of DCV compared to control**	* P-value	**† P-value**
H727	AZ	33 +/− 2.5	p < 0.05	p < 0.01
	SFN	58 +/− 0.8	p < 0.001	p < 0.05
	AZ + SFN	79 +/− 2.3	p < 0.001	-
H720	AZ	24 +/− 1.7	-	p < 0.01
	SFN	48 +/− 2.5	-	p < 0.01
	AZ + SFN	70 +/− 1.8	p < 0.01	-

* P-value was measured by comparing treated tumor to an untreated tumor.

† P-value was measured by comparing single agent treatment to a combination treatment.

### AZ and/or SFN treatment affect the invasive fraction of tumor cells within H-727 xenografts

**Figure 7:**
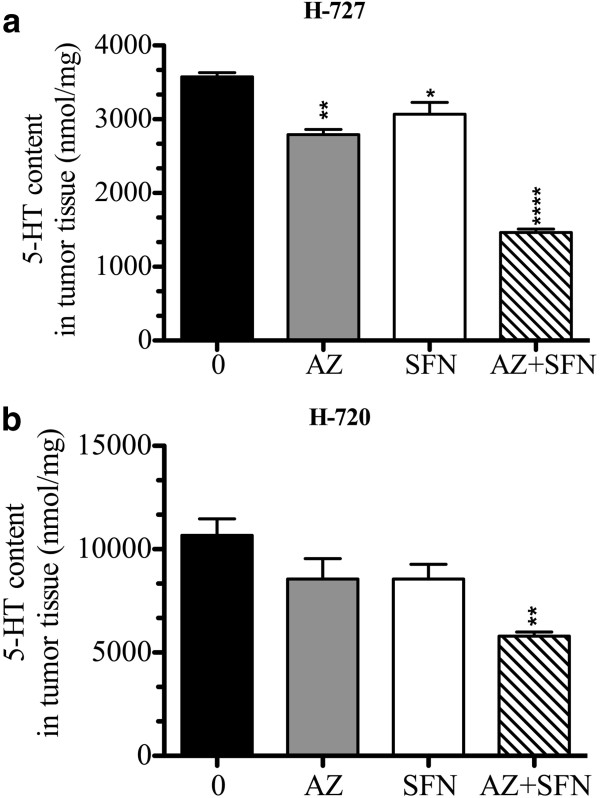
**AZ and SFN Affect Invasive Activity in the H-727 Lung Carcinoid Xenografts: figures (a and b) represent the anti-invasive activity of AZ and/or SFN in H-727 xenografts cells.** Invasive assay was performed in lung carcinoid tumor cells (H-727 xenografts) after treatment of NOD/SCID mice with AZ and/or SFN, 20 mg/kg and/or 40 mg/kg, respectively for 14 days, p < 0.05 (*) and p < 0.0001 (****).

We used the matrigel invasion assay to determine the invasiveness of cells within the xenografts +/− treatments. The fraction of invasive cells was 26%, 39% and 69% for AZ, SFN and AZ + SFN treated tumors compared to untreated group, respectively. The AZ + SFN combination significantly reduced the fraction of invasive cells compared to AZ and SFN (Figure 7a, b), Table 3.

**Table 3:** The percentage of invasive fraction of tumor cells after treatment with AZ and/or SFN within H-727 xenografts

**Cell Type**	**Treatment**	**% Invasive cells fraction**	* P-value	**† P-value**
H727	AZ	26 +/− 6.1	-	p < 0.0001
	SFN	39 +/− 3.2	p < 0.05	p = 0.002
	AZ + SFN	69 +/− 5.3	p < 0.0001	-

* P-value was measured by comparing treated tumor cells to untreated tumor cells.

† P-value was measured by comparing single agent treatment to a combination treatment.

### AZ and/or SFN alone treatment reduced 5-HT content of tumor cells within H-727 and H-720 xenografts

**Figure 8:**
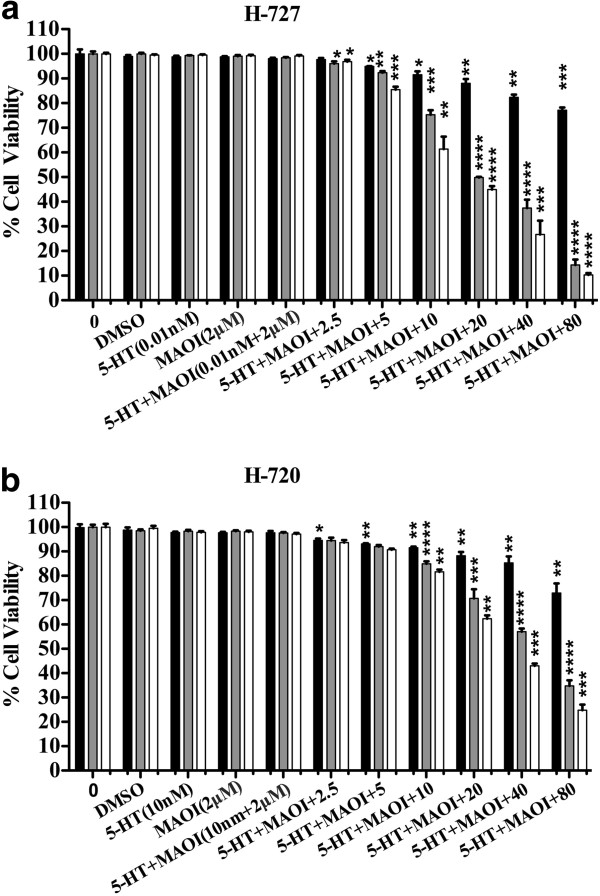
AZ and SFN Treatment Reduce Serotonin Content in the H-727 and H-720 Lung Carcinoid Xenografts: figures (a and b) represent the amount of 5-HT in nmol/mg measured by LC-MS in H-727 and H-720 xenografts; untreated versus treatments for 14 days in lung carcinoid tumor cells after treatment of NOD/SCID mice with AZ and/or SFN, 20 mg/kg and/or 40 mg/kg, respectively; *(p < 0.05), **(p < 0.01), ***(p < 0.001) and ****(p < 0.0001).

The LC-MS assay revealed that all the treatments reduced 5-HT content in the H-727 xenograft model, whereas only the combination caused significant reduction in 5-HT content in H720 xenografts. In H-727 xenografts, compared to the control, AZ, SFN and the combination caused 22%, 14% and 59% reduction in 5-HT content, respectively. In the H-720 model, compared to the control, AZ, SFN and AZ + SFN caused 19%, 19% and 45% reduction in 5-HT content, respectively. Additionally, the combination treatment significantly reduced 5-HT content compared to AZ and SFN treatments for H-727 xenograft cells and SFN treatment for H-720 xenograft cells (Figure 8a, b), Table 4.

**Table 4:** The percentage of 5-HT content of tumor cells within H-727 and H-720 Xenografts after treatment with AZ and/or SFN

**Cell Type**	**Treatment**	**% 5-HT content Compared to Control**	* P-value	**† P-value**
H-727	AZ	22 +/− 1.2	p < 0.001	p < 0.01
	SFN	14 +/− 2.7	p < 0.05	p < 0.05
	AZ + SFN	59 +/− 7.9	p < 0.0001	-
H-720	AZ	19 +/− 9	p < 0.001	p < 0.0001
	SFN	19 +/− 6.2	p < 0.001	p < 0.0001
	AZ + SFN	45 +/− 3.4	p < 0.001	p < 0.0001

* P-value was measured by comparing treated tumor to untreated tumor.

† P-value was measured by comparing single agent treatment to a combination treatment.

### The effect of AZ and/or SFN treatment on 5-HT and growth of lung carcinoid cell lines

**Figure 9:**
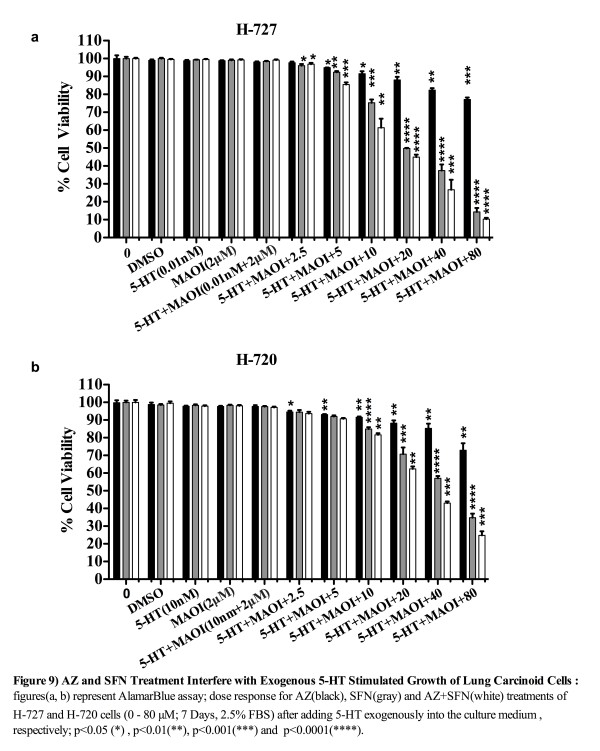
AZ and SFN Treatment Interfere with Exogenous 5-HT Stimulated Growth of Lung Carcinoid Cells: figures (a, b) represent AlamarBlue assay; dose response for AZ (black), SFN (gray) and AZ + SFN (white) treatments of H-727 and H-720 cells (0–80 μM; 7 Days, 205% FBS) after adding 5-HT exogenously into the culture medium, respectively; *(p < 0.05), **(p < 0.01), ***(p < 0.001) and ****(p < 0.0001).

LC-MS measurement proved that FBS contains a considerable amount of 5-HT (9.87 μmol/l). We tested the effect of varying concentrations of FBS (0-20%) on the proliferation of H-727 and H-720 cells to determine the minimum percentage of FBS needed for cell survival for an experiment lasting 7 days. Results showed that the required concentration of FBS for cells to survive for the period of 7 days was 2.5% (data was not shown). We then tested the effect of exogenously added 5-HT in the presence of AZ, SFN and AZ + SFN. As we showed in Figure 9(a, b), lane 1 contained pure cells suspension and lanes 2, 3, 4 and 5 contained cells suspension with vehicle (DMSO), 5-HT (0.01 nM for H-727 and 10 nM for H-720), MAO-AI (2 μM) and 5-HT + MAOI, respectively. Lanes 6–11 contained cells suspension with 5-HT + MAOI that were diluted in the respective cell media and applied in final concentrations (AZ and/or SFN treatment) from 6–11. We found that the AZ + SFN treatment was highly effective in blocking the stimulatory growth effects of 5-HT compared to untreated cells. Importantly, SFN contributed significantly to this inhibition. The minimum concentrations of AZ, SFN and AZ + SFN treatment required to significantly reduce the 5-HT-induced growth effect was 5 μM (2%, p < 0.05), 2.5 μM (4%, p < 0.05) and 2.5 μM (3%, p < 0.05), respectively, for H-727 cells. For H-720 cells, it was 2.5 μM (5%; p < 0.05), 10 μM (15%; p < 0.0001) and 10 μM (18%; p < 0.001) for AZ, SFN and AZ + SFN, respectively. Furthermore, the minimum concentration of combination treatment required to significantly reduce the 5-HT-induced growth effect was 5 μM compared to SFN alone (p = 0.0083) for H-727 cells and 10 μM compared to AZ alone (p = 0.0004) and SFN alone (p = 0.002) for H-720 cells, (Figure 9a, b).

## Discussion

Though carcinoids are slow growing tumors, which can be treated by surgery, the survival in metastatic carcinoids is very low because the treatment strategies for other cancers are not effective for dealing with advanced stage carcinoids [36]. Therefore, the investigations concerning the discovery of new strategies for treating pulmonary carcinoids need to be focused on therapies that can inhibit the growth and invasiveness of advanced stage disease. Carcinoid tumors are proving moderately responsive to newer therapies targeting tumor vasculature and survival pathways [1,2]. The mammalian target of rapamycin (mTOR) inhibitor, everolimus, has shown promising initial results alone or combined with other agents [37-39]. Bronchial AC, which is characterized by high mTOR expression, has been reported to be responders to mTOR inhibition, indicating that therapies targeting the critical survival pathways are potential candidates to treat bronchial carcinoids [40]. The evidence seems to indicate that research for a better therapy for treating BC needs to be focused upon the inhibition of its survival pathways. We believe that AZ and SFN are appropriate drug candidates because of their proven potential to inhibit the survival pathways in other cancers.

High expressions of CAs have been reported in ileal carcinoids [9]. In our original studies, we found that gas sensing (chemo sensing) by pulmonary neuroendocrine cells is an essential function especially in the neonatal period [39]. Furthermore, we learned that lung carcinoid cells produce CAs (manuscript in preparation). AZ is a pan-CA inhibitor which has demonstrated anti-invasive properties against renal cancer cell lines [41]. In other cancers, SFN has demonstrated the potential to inhibit survival pathways, which are also involved in carcinoids. Thus, SFN is reported to affect survival pathway by hyperphosphorylation of Rb protein in colon cancer cells, which is anti-apoptotic in unphosphorylated form [22]. It was shown in previous study that SFN has inhibited cyclin D1 in pancreatic cancer cells [23], while cyclin D1-induced Rb overexpression has been found to be upregulated in pulmonary carcinoids [24]. SFN is also an inhibitor of histone deacetylases (HDAC) [25] and other HDAC inhibitors such as valproic acid and suberoyl bis-hydroxamic acid in combination with lithium have demonstrated significant growth inhibition and cell cycle arrest in H-727 cells [26]. We have showed that SFN alone is effective in inhibiting in vitro and in vivo tumor growth. At higher doses, SFN causes cell cycle arrest and differentiation when used against another aggressive pediatric cancer, neuroblastoma (manuscript in preparation). Therefore, it is reasonable to consider that the combination of AZ and SFN can be investigated for its ability to inhibit the growth and invasive potential of advanced stage carcinoids.

In the present study, both AZ and SFN reduced the viability and clonogenicity of H-727 and H-720 carcinoid cell lines in a dose-dependent manner, in vitro. Both agents delayed tumor growth by reducing the invasive fraction of carcinoid cells and the 5-HT content of tumor. AZ and/or SFN inhibited the autocrine growth effects of 5-HT in a dose-dependent manner. The combination of AZ and SFN demonstrated significant advantage over both as single agents in all respects.

In vitro reduction of viability and clonogenicity of carcinoid cells by both single agents indicates that the significant advantage of combination would be an additive or synergistic effect rather than potentiation. Previously, SFN in combination with cisplatin, gemcitabine, doxorubicin and 5-flurouracil has been reported to reduce the clonogenicity of pancreatic and prostatic cancer cells [42]. Here, the IC50 of AZ and SFN was higher for actively proliferating normal cells FLF, indicating lower susceptibility of normal tissues to our drugs, unlike conventional cytotoxic agents. This could be due to the targeted mechanism of action of our drugs on specific pathways, which are active in carcinoids and are critical for the survival and proliferation of carcinoid cells. PI3K/AKT/mTOR pathway is upregulated in H-727 and H-720 cell lines and these cells have reported to be sensitive to mTOR inhibitors [43-45]. In GI carcinoids, Raf/MEK/ERK pathway is reported to be active. SFN is reported to inhibit Akt/mTor and MEK/ERK/pathways in cancer cells [46,47]. Also, both MEK/ERK and PI3K/AKT pathways are known to regulate the expression of CAIX and these findings might be relevant when combining an inhibitor of CAIX with SFN, which inhibits these pathways [48,49].

The in vivo doses of AZ and SFN were selected on the basis of their efficacies in previous studies. AZ (20 mg/Kg) has demonstrated reduction in spontaneous lung metastasis of lung carcinoma cells at a rate of 62% [50]. In another study, SFN (40 mg/Kg) significantly reduced the tumor weights of orthotopic prostate cancer xenografts compared to untreated control [51]. In our study, in vivo, AZ and SFN demonstrated antitumor efficacy as single agents in both H-727 and H-720 xenografts, while the combination had significantly higher antitumor efficacy in both cases. The in vivo efficacy of AZ and SFN in the mouse subcutaneous xenograft model is in agreement with the in vitro data. In vitro clonogenicity assay has been employed to predict the clinical efficacy of chemotherapeutics. Moreover, the in vitro clonogenicity and invasion assay demonstrates that SFN on it own was more effective overall than AZ on its own. SFN showed greater tumor reduction than AZ. Interestingly, the in vivo results parallel the in vitro results in terms of both the individual and combined drug treatments, which perhaps suggests that the in vitro data may be predictive of the in vivo results.

The indicators of cell death, including condensed nuclei, shrunken cells and apoptotic bodies, observed under the electron microscope in this study, have been used previously to evaluate the apoptotic effect of drug treatment on gastric cancer xenografts [52]. In both H-727 and H-720 xenografts, these effects were more pronounced in the animals treated with the combination. In addition, the electron microscopy results suggest that the combined therapy is more effective at reducing the formation of cytoplasmic dense-core vesicles, which are known to harbor the 5-HT containing granules [53].

Molecule markers such as phospho-histone 3, Ki67 and ChA and TPH were used to examine the antitumor effectiveness of treatment(s) on H-727 and H-720 xenograft models. pHH3 serves as a marker of mitosis and was used to determine the mitotic index in H-727 and H-720 xenografts [54]. The mitotic index was significantly reduced in all groups compared to the control. The combination treated mice had a significantly lower mitotic index compared to either AZ or SFN treated mice. Ki67, the proliferation marker, is associated with low survival in patients with lung cancers, including TC and AC [55]. We found that the proliferative index did not change although the Ki67 staining intensity appeared greater in all the treated animals. This might be expected of cells that are arrested in the cell cycle since Ki67 is expressed in all phases (variably) but not in G0. In the present study, the reduction in the levels of ChA upon treatment with AZ and/or SFN indicates the antiserotonergic nature of the therapy [56].

After invasive assay, the cells that were characterized as invasive were counted. These were then cultured and passaged three times and stained with specific lung carcinoid marker (ChA) to confirm that the invasive cells were originated from tumor cells and not the non-cellular component of xenografts. The invasive H-727 xenograft cells phenotypically matched with H-727 cells in monolayer culture with positive expression of ChA in these cells. We observed that SFN caused reduction in the invasive potential of cells isolated from H-727 xenografts, an effect which was significantly enhanced by the combination. Although AZ alone did not affect the invasiveness of H-727 cells, it potentiated the anti-invasive property of SFN. This finding is in agreement with previous reports where SFN inhibited the in vitro migration of oral carcinoma cells by down regulation of MMP-1 and MMP-2 secretion and ovarian cancer cells by increasing apoptotic cell death via an increase in Bak/Bcl-2 ratio and cleavage of procaspase-9 and poly (ADP-ribose)-polymerase (PARP) [57,58]. Since the 5-year survival rate in metastatic bronchial carcinoids is only 20-30% [4], reduction in the invasive carcinoid cell population upon in vivo AZ + SFN treatment indicates its possible advantage in treating metastatic disease.

Since AZ and SFN can reduce the number of viable carcinoid cells, we hypothesized that the treatment could affect 5-HT content of the tumor. We observed a reduction in 5-HT content of tumor xenografts following the treatment with AZ and/or SFN. The reduction of TPH expression as observed by IHC corroborates with the reduction in 5-HT levels and provides an additional possible mechanism by which AZ and/or SFN reduce 5-HT levels. Inhibition of TPH as a means to reduce 5-HT levels has been used in the case of LX1031, a novel drug being investigated for managing carcinoid syndrome [59]. However, no agent reducing TPH expression has been reported for managing carcinoid syndrome. The mechanism by which our drugs reduce TPH expression can be speculated on the basis of previous reports. HDAC has been implicated in the reduction of TPH expression in mood disorder patients [60,61]; therefore, HDAC inhibition by SFN may have caused TPH reduction. Several factors can contribute to the synergistic effect on 5-HT reduction, including increased apoptosis of 5-HT producing carcinoid cells and the effect of CA inhibition on 5-HT production. Moreover, AZ and/or SFN reduced 5-HT-induced in vitro proliferation of carcinoid cells in the present study. Reduction in 5-HT content of the tumor and the inhibition of 5-HT-mediated autocrine growth effects can be two possible mechanisms contributing to increased antitumor efficacy by the combination and can also manage carcinoid syndrome.

## Conclusion

We show for the first time that the growth of bronchial carcinoids is significantly inhibited in vitro and in vivo by AZ and/or SFN treatment in a dose-dependent relationship. Furthermore, AZ and/or SFN treatment caused a reduction in 5-HT content of the carcinoid cells both in vitro and in vivo. The combination of the two agents produced a more marked and efficacious effect than did a single agent. Since the effective doses of single agents and the combination are well within clinical range and bioavailability, our results suggest a potential new therapeutic strategy for the treatment of bronchial carcinoids.

## Abbreviations

5-HIAA: 5-Hydroxyindole-3-acetic acid; 5-HT: 5 hydroxytryptamine; AZ: Acetazolamide; TC: Typical carcinoid; AC: Atypical carcinoid; pHH3: Phospho-Histone H3; CAs: Carbonic anhydrases; ChA: Chromogranin A; DCV: Dense-core vesicles; SFN: Sulforaphane; TPH: Tryptophan hydroxylase.

## Competing interests

The author(s) declare that they have no competing interests.

## Authors’ contributions

RBM: initiated the study, designed the experimental approach, conducted the experiments, and analyzed the data; SK and SSI: assisted with the in vitro and in vivo experiments; MY: performed LC-MS for 5-HT; HY: conceived the study idea, initiated and supervised the study; RBM and SK wrote the original manuscript; HY, KA and EC edited the manuscript. All authors read and approved the final manuscript.

## Pre-publication history

The pre-publication history for this paper can be accessed here:

http://www.biomedcentral.com/1471-2407/13/378/prepub
